# Blood Flow Restriction Increases the Neural Activation of the Knee Extensors During Very Low-Intensity Leg Extension Exercise in Cardiovascular Patients: A Pilot Study

**DOI:** 10.3390/jcm8081252

**Published:** 2019-08-19

**Authors:** Hayato Ishizaka, Azusa Uematsu, Yuta Mizushima, Naohiro Nozawa, Satoshi Katayanagi, Kazuhisa Matsumoto, Kaori Nishikawa, Reiko Takahashi, Tomoe Arakawa, Tatsuya Sawaguchi, Tomohiro Yasuda, Suomi Yamaguchi, Hironaga Ogawa, Ikuko Shibasaki, Shigeru Toyoda, Tibor Hortobágyi, Hirotsugu Fukuda, Teruo Inoue, Takashi Mizushima, Toshiaki Nakajima

**Affiliations:** 1 Department of Rehabilitation, Dokkyo Medical University Hospital, Shimotsuga-gun, Tochigi 321-0293, Japan; 2 Department of Health and Sport Sciences, Premedical Sciences, Dokkyo Medical University, Shimotsuga-gun, Tochigi 321-0293, Japan; 3 Department of Cardiovascular Medicine, School of Medicine, Dokkyo Medical University, Shimotsuga-gun, Tochigi 321-0293, Japan; 4 School of Nursing, Seirei Christopher University, Hamamatsu, Shizuoka 433-8558, Japan; 5 Department of Cardiology and Nephrology, School of Medicine, Dokkyo Medical University, Shimotsuga-gun, Tochigi 321-0293, Japan; 6 Department of Cardiac and Vascular Surgery, School of Medicine, Dokkyo Medical University, Shimotsuga-gun, Tochigi 321-0293, Japan; 7 University Medical Center Groningen, University of Groningen, Groningen, Groningen 9713 GZ, The Netherlands; 8 Department of Rehabilitation, School of Medicine, Dokkyo Medical University, Shimotsuga-gun, Tochigi 321-0293, Japan

**Keywords:** cardiovascular patient, leg extension, electromyographic activity, blood flow restriction

## Abstract

Blood flow restriction (BFR) has the potential to augment muscle activation, which underlies strengthening and hypertrophic effects of exercise on skeletal muscle. We quantified the effects of BFR on muscle activation in the rectus femoris (RF), the vastus lateralis (VL), and the vastus medialis (VM) in concentric and eccentric contraction phases of low-intensity (10% and 20% of one repetition maximum) leg extension in seven cardiovascular patients who performed leg extension in four conditions: at 10% and 20% intensities with and without BFR. Each condition consisted of three sets of 30 trials with 30 s of rest between sets and 5 min of rest between conditions. Electromyographic activity (EMG) from RF, VL, and VM for 30 repetitions was divided into blocks of 10 trials and averaged for each block in each muscle. At 10% intensity, BFR increased EMG of all muscles across the three blocks in both concentric and eccentric contraction phases. At 20% intensity, EMG activity in response to BFR tended to not to increase further than what it was at 10% intensity. We concluded that very low 10% intensity exercise with BFR may maximize the benefits of BFR on muscle activation and minimize exercise burden on cardiovascular patients.

## 1. Introduction

Cardiovascular patients frequently present low levels of cardiovascular function, muscle mass, and muscle strength. Aerobic and resistance exercise as part of cardiac rehabilitation can reduce these deficits [1,2]. Because high-intensity exercise increases the risk of arrhythmias and can exacerbate existing heart failures [3], the intensity of aerobic exercise is kept below anaerobic threshold during cardiac rehabilitation [4]. During conventional cardiac rehabilitation, patients perform aerobic exercise on ergometers or treadmills, and exercise intensity is set based on cardiopulmonary exercise testing. Indeed, light intensity aerobic exercise tends to improve cardiopulmonary function, but this intensity is insufficient to improve muscle strength and mass [5]. Therefore, light-intensity exercise programs, which can also improve muscle strength and mass, are needed to safely facilitate cardiovascular patients’ rehabilitation.

Short-term, low-intensity exercise with blood flow restriction (BFR) versus exercise without BFR of a limb muscle increases the gains in muscle strength and mass in athletes and healthy adults [6,7]. The potential physiological mechanism underlying low-intensity exercise with BFR to improve muscle strength and mass is an increase in metabolic stress, which theoretically activates systemic hormone production and fast-twitch muscle fibers [8]. A clinical study also reported that low-intensity resistance training with BFR can increase lower limb muscle strength and mass in cardiovascular patients [9]. However, the effects of BFR on muscle activation during low-intensity exercise in cardiovascular patients are still unknown. Because exercise with BFR vs. exercise without BFR has the potential to augment muscle activation, which can be recorded noninvasively by electromyography (EMG), it is relevant to determine the effects of BFR on EMG activity in cardiovascular patients.

The purpose of this pilot study was to determine the effects of BFR on the neural activation of the knee extensors during low-intensity exercise in cardiovascular patients. Because an increase in metabolic stress is the main trigger to facilitate muscle activation in healthy adults [8], we hypothesized that BFR can also increase muscle activation in cardiovascular patients. In addition, we sought to determine if BFR differentially activates synergistic muscles. Because BFR increases muscle activation more during concentric than eccentric contractions in healthy young adults [10], we were also interested in determining if muscle activation occurs also in a contraction-specific manner when BFR is applied in cardiovascular patients. Therefore, we examined the effects of BFR on EMG activation of the rectus femoris (RF), the vastus lateralis (VL), and the vastus medialis (VM) during low-intensity leg extension exercise in cardiovascular patients.

## 2. Methods

### 2.1. Participants

This study used a quasi-experimental design. Seven cardiovascular patients (6M, Table 1) participated in this pilot study. Of the 15 cardiovascular patients who participated in outpatient cardiac rehabilitation between July 2018 and November 2018, seven patients met the following criteria: (1) completion of postoperative cardiac rehabilitation consisting of aerobic exercise, (2) ability to perform the pre-test of 1 repetition maximum (RM) leg extension and leg extension exercise at 10–20% of 1 RM, and (3) consent to participating in the study. Patients visited the hospital’s cardiac rehabilitation clinic. A cardiologist medically cleared and declared each patient fit for the experiment. Participants gave written informed consent prior to testing. The University Ethics Committee approved the study protocol, which was conducted according to Declaration of Helsinki (ID 27074/2015, date of approval: 13th October, 2015).

Table 1 shows the patients’ characteristics and the drugs prescribed.

### 2.2. Experimental Protocol

Participants performed bilateral leg extensions on a leg extension machine (GX-320, OG Wellness, Co. Ltd, Okayama, Japan) in a seated position, starting the exercise with hips and knees flexed at 90° and at 95°, respectively. During exercise, participants grasped the handlebars on the side of the leg extension machine and performed leg extensions from the flexed starting position to full knee extension. At least 3 days before the experiment, we measured participants’ 10 RM and estimated the leg extension 1 RM [11]. In a preliminary experiment, some of the patients could not lift a load heavier than 30% of 1 RM with BFR, thus we selected exercise loads of 10% (very low-intensity) and 20% intensities (low-intensity).

**Figure 1:**
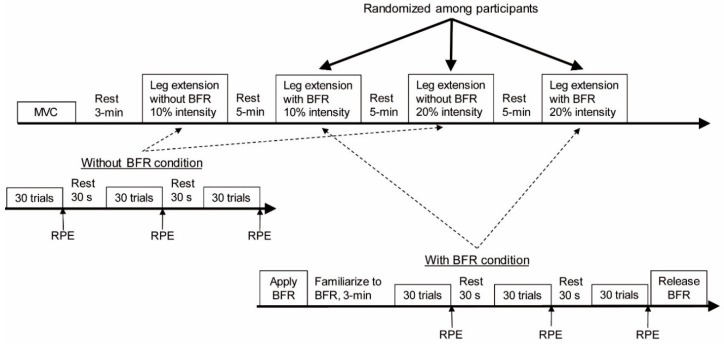
Experimental protocol. Upper part depicts the experimental timeline. Middle part details exercise without blood flow restriction (BFR), and lower part details the exercise with BFR.

Before exercise, participants performed a maximal voluntary isometric knee extensor contraction (MVC) in the starting position on the leg extension machine. They performed leg extensions under 4 conditions: 10 and 20% intensities with or without BFR. In each condition, they completed 3 sets of 30 trials of bilateral knee extensions with 30 s of rest between sets and 5 min of rest between conditions. A technician counted the number of repetitions paced by a metronome at 60 beats per minute. The experiment started with the 10% intensity without BFR. The other 3 conditions were block-randomized among participants. A medical doctor was present at all times and monitored each participant performing the exercise. Figure 1 shows the experimental protocol.

One participant (No. 7) was unable to complete the 20% intensity with BFR. Therefore, 7 participants completed 10% intensity, and 6 participants completed both 10% and 20% intensities.

### 2.3. Blood Flow Restriction

We used a compact KAATSU system (KAATSU Nano, KAATSU Global, Huntington Beach, CA, USA) to artificially restrict blood flow in the thigh. The pneumatic cuff (60 mm wide, the KAATSU Air Bands, KAATSU Global, Huntington Beach, CA, USA) was placed around the proximal end of both thighs while participants were seated on the leg extension machine. We set the pressure to 180 mmHg for BFR in the lower limbs because such moderate BFR can minimize a loss of muscle function during exercise and still produce a training effect [12]. We applied BFR during leg extension, and the cuff remained inflated during the 30 s rest between sets. BFR was released after the completion of each exercise condition.

### 2.4. Data Collection

We recorded surface EMG activity from the right rectus femoris (RF), the vastus lateralis (VL), and the vastus medialis (VM) using active surface electrodes (2 mm width, 10 mm length, 10 mm between electrodes, SS-2096, Nihon Kohden, Tokyo, Japan). The skin was cleaned with alcohol-soaked cotton to reduce skin impedance. The earth electrode was affixed to skin over the right anterior superior iliac spine. The EMG signals were transmitted (ZB-581G, Nihon Kohden, Tokyo, Japan) to a receiver (ZR-550H, Nihon Kohden, Tokyo, Japan) connected to a multi telemeter system (WEB-5500, Nihon Kohden, Tokyo, Japan). A goniometer (SG 150, Biometrics, Newport, UK) was attached to the lateral side of the left knee to measure range of motion. All signals were digitized at 2 kHz using the multi telemeter system. The EMG signals were band-pass filtered (15–500 Hz), and the goniometer signal was low-pass filtered at 6 Hz (Spike 2, Cambridge Electronics Devices, Cambridge, UK).

We monitored exercise intensity by asking patients to report their subjective ratings of perceived exertion (RPE) on a Borg scale of 6 to 20 [13] after each set.

### 2.5. Data Analysis

Based on the goniometer signal, each trial was divided into concentric and eccentric contraction phases. After subtracting the direct current (DC) component, the EMG signals were full-wave rectified and averaged for each concentric and eccentric contraction phase. To compare the EMG amplitude between concentric and eccentric contraction phases, we averaged the EMG data for all trials and normalized as %MVC. To examine the relative EMG changes in concentric and eccentric contraction phase, we divided the 30 trials into 3 blocks consisting of 10 trials. The EMG data were averaged for each block and normalized by the mean EMG value computed for the initial 10 contractions of the first exercise set at 10% intensity without BFR. PRE was averaged for 3 sets in each condition.

### 2.6. Statistics

All data are presented as mean ±SE. The main analysis was Time (1–10, 11–20, 21–30 contraction blocks) by Load (10%, 20% intensities) by BFR (with, without BFR) by Muscle (RF, VL, VM) mixed design ANOVA for each set. When ANOVA revealed a significant interaction or main effect including the BFR factor, Bonferroni’s multiple comparison was performed to determine the effects of BFR on muscle activations. We also determined the relationship between age and EMG activation and age and RPE. The level of significance was set at *p* < 0.05.

## 3. Results

### 3.1. Electromyographic Activity During Concentric and Eccentric Contractions

Table 2 summarizes average EMG amplitude data in concentric and eccentric contraction phases. There was a Phase by Load by BFR interaction (*F* = 22.1, *p* < 0.001). Because ANOVA did not detect any interactions including the Muscle factor, we averaged EMG activity for RF, VL, and VM. EMG amplitude in the concentric vs. the eccentric contraction phase was greater at the same load and BFR condition (all, *p* < 0.001).

### 3.2. Relative Electromyographic Changes in the Concentric Contraction Phase

**Figure 2:**
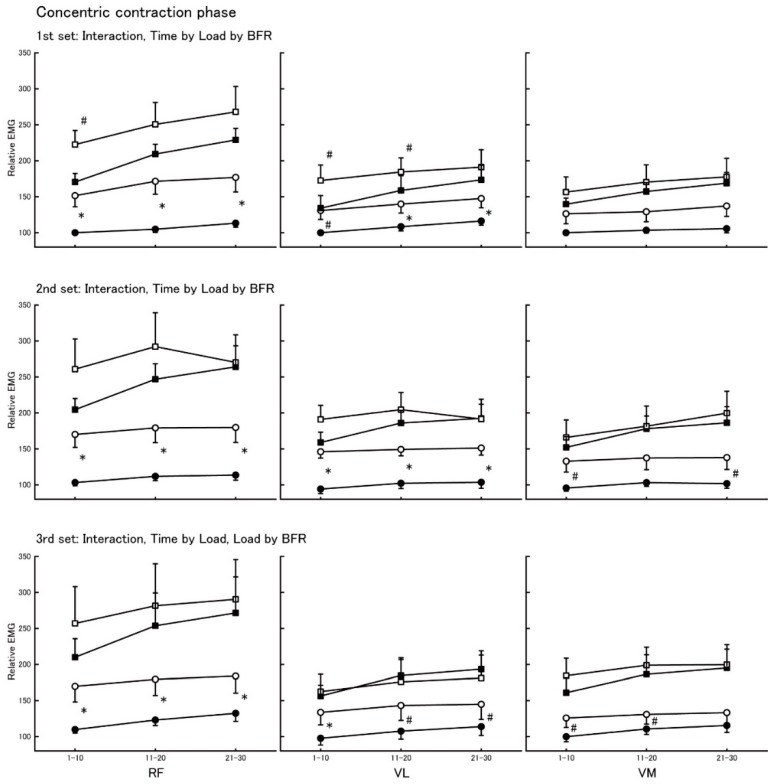
Relative EMG activity in the concentric contraction phase. Upper panels: first set; middle panels: second set; lower panels: third set; left panels: data of RF; center panels: data of VL; right panels: data of VM. Filled circle: 10% intensity without BFR; open circle: 10% intensity with BFR; filled square: 20% intensity without BFR; open square: 20% intensity with BFR. * denotes significance (*p* < 0.05), and # denotes a trend (*p* < 0.10) of EMG increase by adding BFR at same intensity.

Figure 2 shows the relative EMG of RF, VL, and VL in the concentric contraction phase. There was a Time by Load by BFR interaction for the first (*F* = 6.9, *p* = 0.003) and the second set (*F* = 4.5, *p* = 0.021). For the third set, ANOVA detected a Time by Load (*F* = 5.9, *p* = 0.008) and a Load by BFR (*F* = 13.7, *p* = 0.003) interaction.

For 10% intensity, BFR increased relative EMG in RF and VL at all time points (Figure 1, left and middle panels) and tended to increase in VM (Figure 1, right panels) in each set. For 20% intensity, BFR tended to increase the relative EMG in RF, VL, and VM but did not reach significance or a trend at most time points.

### 3.3. Relative Electromyographic Changes in the Eccentric Contraction Phase

**Figure 3:**
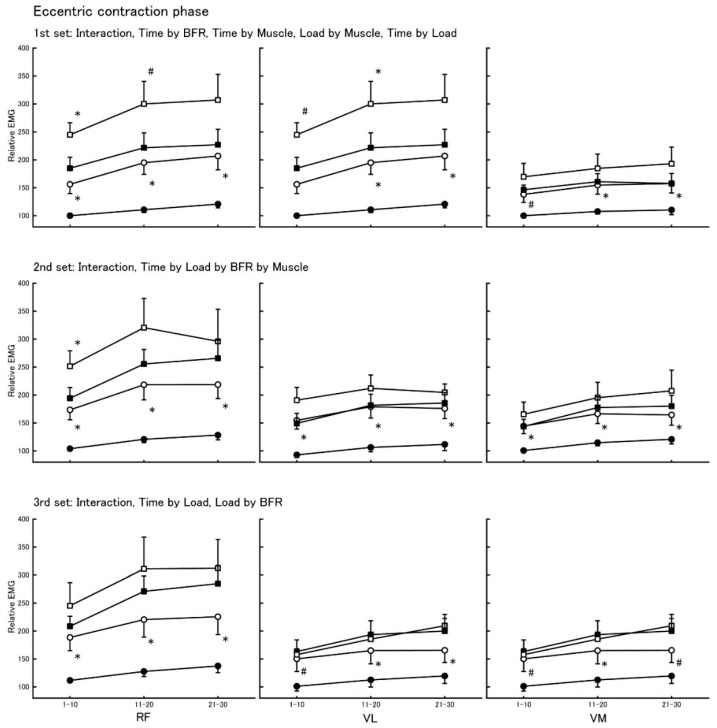
Relative EMG activity in the eccentric contraction phase. Upper panels: first set; middle panels: second set; lower panels: third set; left panels: data of RF; center panels: data of VL; right panels: data of VM. Filled circle: 10% intensity without BFR; open circle: 10% intensity with BFR; filled square: 20% intensity without BFR; open square: 20% intensity with BFR. * denotes significance (*p* < 0.05), and # denotes a trend (*p* < 0.10) of EMG increase by adding BFR at same intensity.

Figure 3 shows the relative EMG of RF, VL, and VM in the eccentric contraction phase. In the first set, there were Time by BFR (*F* = 7.6, *p* = 0.002) and Time by Muscle interactions (*F* = 4.4, *p* = 0.007) and trends for Load by Muscle (*F* = 3.4, *p* = 0.061) and Time by Load interactions (*F* = 3.2, *p* = 0.055). In the second set, there was a Time by Load by BFR by Muscle interaction (*F* = 3.4, *p* = 0.026). In the thirrd set, there was a Time by Load interaction (*F* = 14.94, *p* < 0.001) and a trend for Load by BFR interaction (*F* = 3.8, *p* = 0.075).

For 10% intensity, BFR increased relative EMG in RF, VL, and VL at most time points (Figure 2). For 20% intensity, BFR increased relative EMG in RF and VL in the first set (Figure 2, upper panels), but the effects of BFR on relative EMG tended to decrease in the second and the third set in this order (Figure 2, second upper panels and lower panels).

### 3.4. Subjective Exercise Intensity

Table 3 shows the RPE data. RPE increased, in order: (1) 10% intensity without BFR; (2) with BFR (+1.9 RPE units increase); (3) 20% intensity without BFR (+1.6 RPE units increase); and (4) with BFR (+1.5 RPE units increase).

### 3.5. Correlations Between Age and Relative EMG and RPE in the BFR Condition

Age correlated with relative EMG in the eccentric contraction phase at 20% intensity (*r* = 0.90, *p* < 0.05) in the first set, the eccentric contraction phase at 10% (*r* = 0.77, *p* < 0.05) and 20% intensity (*r* = 0.96, *p* < 0.01) in the second set, and the concentric contraction phase at 10% intensity (*r* = 0.79, *p* < 0.05) and the eccentric contraction phase at 10% (*r* = 0.82, *p* < 0.05) and 20% intensity (*r* = 0.93, *p* < 0.05) in the third set. There were no significant correlations between age and RPE in the BFR conditions.

## 4. Discussion

We examined the effects of BFR on the neural activation of the knee extensor muscles during concentric and eccentric contraction phases of leg extension exercise at 10% and 20% intensity of 1 RM in cardiovascular patients. We found that BFR compared with no BFR increased the activation of RF, VL, and VM at the 10% intensity, but the BFR-induced increases in EMG tended to decrease at the 20% intensity in both concentric and eccentric contraction phases of the knee extension exercise.

Previous studies reported that BFR increased muscle activation during low (20% of 1 RM) [14,15,16] and moderate (40% of 1 RM) activity [7] but did not increase [7] or even decreased [17] muscle activation during high-intensity (70% of 1 RM) contraction in healthy young adults. The high-intensity contraction might have restricted blood flow, and the addition of BFR could not further compress the blood vessels, producing no additional changes in EMG activity [17]. Previous studies also noted that BFR could reduce the number of muscle contraction (30% of 1 RM) to task failure [18], and BFR-induced increases in EMG activity tended to decrease with increasing number of muscle contraction at low-intensity (20% of 1 RM) exercise [15]. Together, these previous studies suggest that high-intensity or fatiguing exercise minimizes BFR benefits for muscle activation. Therefore, the BFR-induced increase in muscle activation can be maximized with low-intensity and non-fatiguing muscle contractions. The present data complement these previous data in healthy adults by showing that BFR-induced increases in muscle activation tended to decrease at the 20% intensity in our patients with cardiovascular disease (Figure 2 and Figure 3). A recent review suggested that heart failure patients develop skeletal muscle fatigability and exercise intolerance, especially in the lower extremities [19]. In support of this possibility, we observed higher RPE at 20% vs. 10% intensity (Table 3). The BFR-induced increases in muscle activation can plateau at 20% intensity in cardiovascular patients so that patients perform fewer contractions than healthy young adults [15]. In fact, the female patient (No. 7) with congestive heart failure (CHF) and low left ventricular ejection fraction (LVEF) could not perform leg extension exercise at 20% intensity with BFR, suggesting that muscle fatigability and exercise intolerance in the lower extremities [19] had occurred already at 20% intensity with BFR.

EMG activation was lower during eccentric compared with concentric muscle contractions at the same absolute load [20,21]. We observed a similar pattern in our cardiovascular patients’ RF, VL, and VM (Table 2). The lower EMG activation during eccentric compared with concentric contraction is probably related to the contribution of passive viscous element to force, elements which are neurally inert [22]. Motor units also discharge action potentials at a lower rate during eccentric vs. concentric contractions [23]. Considering these contraction-specific differences, the effects of BFR on muscle activation may be also contraction-specific. In healthy young adults, muscle activation strongly increases when muscles are contracted concentrically vs. eccentrically with BFR [10]. In contrast, results of the present study showed that BFR increased EMG activity in RF, VL, and VM at 10% intensity, but the augmenting effect of BFR at 20% intensity in both concentric and eccentric contractions diminished (Figure 2 and Figure 3). We speculate that cardiovascular disease compromises the mechanical function of patients’ atrophic skeletal muscle to resist the external load, which requires a compensatory increase in the number of muscle fibers recruited during eccentric contraction compared with healthy young adults. Thus, there is a possibility that BFR, which induces metabolic changes in energy supply [24,25], venous oxygen saturation, partial pressure of oxygen and carbon dioxide, and accumulation of lactate and hydrogen ions [26], similarly increases muscle activation during both concentric and eccentric contraction in cardiovascular patients who often present with muscle fatigability and exercise intolerance due to muscle atrophy. To clarify this possibility, future studies will examine the relationship between muscle mass and magnitude of BFR-induced increase of muscle activation.

The source of skeletal muscle fatigability in heart failure patients is presumably related to changes in skeletal muscle metabolism, an impaired ability of muscle fibers to become active in response to the motor command, and low muscle mass and strength [19]. BFR induces metabolic changes [7,24,25] and increases muscle activation [7,14,15,16] and skeletal muscle hypertrophy [27]. Taken together, exercise with BFR has many benefits and could accelerate cardiac patients’ rehabilitation. However, here we show, for the first time, that BFR compared with no BFR increased knee extensor muscle activations at an exercise intensity of only 10%. In addition, RPE of 14.2 at 20% intensity without BFR and 15.7 at 20% intensity with BFR exceeded RPE 13, which denotes a moderate perceived intensity. Therefore, we recommended starting cardiovascular patients’ rehabilitation with BFR at a very low 10% intensity, a process that would still maximize muscle activation and keep the exercise load patients perceive low. Such a BFR exercise program will be applied to patients with CHF and low LVEF. We have preliminary indications that, under such conditions, patients can experience substantial muscle hypertrophy (unpublished data). Indeed, future studies will examine in detail the chronic effects of BFR on muscle mass, muscle metabolism, and muscle fatigability in patients undergoing cardiac rehabilitation.

One limitation of the present pilot study is the low sample size. A second limitation is the diversity of clinical diagnoses, age (25–70 years), and the inclusion of only one female patient. However, the correlation analyses suggest that older compared with younger patients may benefit more from the BFR-induced increases in muscle activation at a similar perceived exercise load. Also, the trends were similar in the six male patients and the one female patient, but the effect of sex on the benefits of BFR will have to be further examined. In addition, most participants had medical treatment [β-blocker, calcium channels blockers (CCB), angiotensin converting enzyme inhibitor (ACEI), angiotensin II receptor blocker (ARB)], and the interactions between drugs and BFR treatment are unknown. We attempted to address this limitation by comparing the effects of exercise with and without BFR on muscle activation on the same day, suggesting minimal drug effects. Clearly, further studies are needed to examine whether the present results are representative of the general population of cardiovascular patients.

## 5. Conclusions

We demonstrated that BFR increased muscle activation similarly in individual knee extensor muscles at very low-intensity (10% intensity) knee extension, but this effect reached a ceiling at low-intensity (20% intensity). We recommend starting cardiovascular patients’ rehabilitation with BFR at a very low 10% intensity, a process that would still maximize muscle activation and keep patient burden low.

## Figures and Tables

**Table 1:** Patient characteristics.

Patient No, Sex	Age (years)	Height (cm)	Weight (kg)	LVEF	Drugs	Diagnosis	Days after Diagnosis
No 1, Male	42	168.0	57.5	66%	ACEI, β-blocker	Post MVP	482
No 2, Male	25	168.0	61.3	53%		Post AVR	105
No 3, Male	44	172.0	77.5	52%	ARB, β-blocker	Post AVR, TAP	482
No 4, Male	66	170.7	68.0	20%		Post AVR, TAP	476
No 5, Male	70	171.4	67.5	46%	CCB, ACEI, β-blocker	Post BO	293
No 6, Male	46	170.0	67.0	63%		Post AVR, TAP	187
No 7, Female	43	158.0	49.2	37%	β-blocker	CHF, MR, AR	1018

LVEF, left ventricular ejection fraction; ACEI, angiotensin converting enzyme inhibitor; β-blocker, β-blocking agents; MVP, mitral valve plasty; AVR, aortic valve replacement; ARB, angiotensin II receptor blocker; TAP, tricuspid annuloplasty; CCB, calcium channels blockers; BO, Bentall operation; CHF, congestive heart failure; MR, mitral regurgitation; AR, aortic regurgitation.

**Table 2:** Electromyographic activity (EMG) activation amplitude during concentric and eccentric contraction phase of leg extension exercise.

	10% Intensity				20% Intensity			
	Without BFR		With BFR		Without BFR		With BFR	
	CON	ECC	CON	ECC	CON	ECC	CON	ECC
RF	10.9 (1.8)	7.7 (1.0)	15.4 (2.2)	12.6 (1.8)	18.4 (2.5)	13.5 (1.6)	19.5 (2.3)	15.8 (2.0)
VL	15.1 (2.1)	10.2 (1.1)	20.3 (2.6)	15.5 (2.2)	23.6 (3.2)	16.5 (2.3)	25.2 (3.2)	18.3 (1.9)
VM	12.6 (1.6)	9.3 (1.3)	15.6 (2.0)	12.4 (1.7)	20.2 (3.0)	14.3 (2.2)	20.2 (3.0)	15.8 (2.3)
AVG	11.8 (2.0) *	8.5 (1.1)	16.2 (2.4) *	12.7 (1.9)	20.3 (3.2) *	14.2 (2.1)	21.6 (3.2) *	16.6 (2.3)

Data are mean (±SE) as a percent of maximal voluntary contraction (%MVC); BFR, blood flow restriction; CON, concentric contraction phase; ECC, eccentric contraction phase; RF, rectus femoris; VL, vastus lateralis; VM, vastus medialis; AVG, average EMG value for RF, VL, and VM; *, EMG amplitude is higher during concentric vs. eccentric contraction at same intensity in same BRF condition (all, *p* < 0.001).

**Table 3:** Ratings of perceived exertion (RPE) after each exercise condition.

	10% Intensity		20% Intensity	
	Without BFR	With BFR	Without BFR	With BFR
RPE	10.7 (0.2)	12.6 (0.5) ^†^	14.2 (0.4) ^††^	15.7 (0.7) ^†††^

Data are mean (±SE); RPE, rate of perceived exertion; BFR, blood flow restriction; ^†^, RPE is higher with BFR vs. without BFR at 10% intensity (*p* < 0.001); ^††^, RPE is higher without BFR at 10% intensity vs. with BFR at 10% intensity (*p* < 0.001); ^†††^, RPE is higher with BFR vs. without BFR at 20% intensity (*p* < 0.001).
